# The cerebellar involvement in essential tremor: the connecting roads

**DOI:** 10.1055/s-0045-1812324

**Published:** 2025-10-27

**Authors:** Carlos Henrique Ferreira Camargo, Léo Coutinho, Luís Eduardo B.M. Zubko, Gustavo L. Franklin, Hélio Afonso Ghizoni Teive

**Affiliations:** 1 Universidade Federal do Paraná, Programa de Pós-Graduação em Medicina Interna, Disciplina de Doenças Neurodegenerativas, Curitiba PR, Brazil.; 2 Universidade Federal do Paraná, Departamento de Clínica Médica, Serviço de Neurologia, Curitiba PR, Brazil.; 3 Universidade Federal do Paraná, Departamento de Clínica Médica, Serviço de Clínica Médica, Curitiba PR, Brazil.; 4 Pontifícia Universidade Católica do Paraná, Faculdade de Medicina, Departamento de Clínica Médica, Curitiba PR, Brazil.

**Keywords:** Essential Tremor, Cerebellum, Purkinje Cells, Inferior Olivary Complex, Cholinergic Antagonists

## Abstract

Essential tremor (ET) is the most prevalent movement disorder globally, affecting about 1% of the general population and 5% of those aged over 65 years. Characterized by involuntary, rhythmic oscillations, it primarily manifests as postural and kinetic tremors, predominantly in the upper limbs. Genetic studies, neuropathological examinations, neurophysiological assessments, and various neuroimaging techniques have demonstrated functional, neurotransmitter-related, and structural abnormalities within the cerebello-thalamo-cortical circuit. These findings collectively portray ET as a neurodegenerative syndrome with diverse etiologies and clinical manifestations, highlighting the involvement of the cerebellum.

## INTRODUCTION

Essential tremor (ET) is the most prevalent movement disorder globally, affecting about 1% of the general population and 5% of those over 65. 1 Characterized by involuntary, rhythmic oscillations, ET primarily manifests as postural and kinetic tremors, predominantly in the upper limbs, with a frequency ranging from 4 to 12 Hz. While it traditionally affects the hands, in approximately 95% of the cases, other areas can also be involved, such as the head, tongue, legs, voice, face, chin, and torso. 2

Historically, ET was first differentiated from Parkinson's disease (PD) by James Parkinson in 1817, a distinction published posthumously in 1872 by Jean-Matin Charcot. 3 4 5 The term *essential tremor* (initially from the Italian “tremore semplice essenziale”) was coined by Pietro Burresi in 1874 and, over the years, the understanding of ET has evolved significantly. 6 Once considered a monosymptomatic condition, recent studies have challenged this view, documenting additional motor signs and a range of nonmotor features, thus recognizing it as a complex neurodegenerative syndrome with diverse causes and manifestations. 7 8

Neuroimaging and functional studies have played a crucial role in enhancing our understanding of ET, particularly highlighting the cerebellum's involvement. These studies have demonstrated structural and functional abnormalities in this area, associated with altered connectivity within the cerebello-thalamo-cortical circuit. This disrupted connectivity is evident in reduced links between the primary motor cortex and the cerebellum, as well as heightened connectivity between the thalamus and the cerebellum, suggesting the its central role in tremor generation through the modulation of central oscillators. 9 10 11

The aim of this narrative review is to synthesize and organize the findings from various studies concerning the pathophysiology, neuropathology, genetics, clinical manifestations, neuroimaging, and treatment approaches that highlight the cerebellum's crucial role in the genesis of ET.

## METHODS

The current narrative review encompassed original research articles, including observational, cohort, cross-sectional, and case-control studies, as well as case series, clinical cases, metanalysis, and reviews that contribute to connecting genetics, clinical findings, neurophysiology, neuroimaging, and the pathophysiology of ET with the cerebellum.

A thorough three-step search strategy was employed to gather relevant literature:

- Initial search: A preliminary exploration was conducted in key databases such as PubMed, Embase, and CINAHL using terms like *essential tremor* and *cerebellum* . This step involved retrieving and identifying index terms, Medical Subject Headings (MeSH), and keywords from the title and abstract of relevant papers;
- Full search: This subsequent, more extensive search incorporated all the identified keywords and index terms across the aforementioned databases;
- Hand search: The reference lists of all located studies were manually searched to uncover additional ones that might not have been included in the database searches.

The references from the articles were also thoroughly searched for additional articles.

Two reviewers independently screened the literature for each topic: pathophysiology, pathology, neurophysiology (CHFC and GLF), as well as clinical alterations and neuroimaging (LEBMZ and LC). Any discrepancies between reviewers were resolved through discussion.

## PATHOPHYSIOLOGY

**Figure 1:**
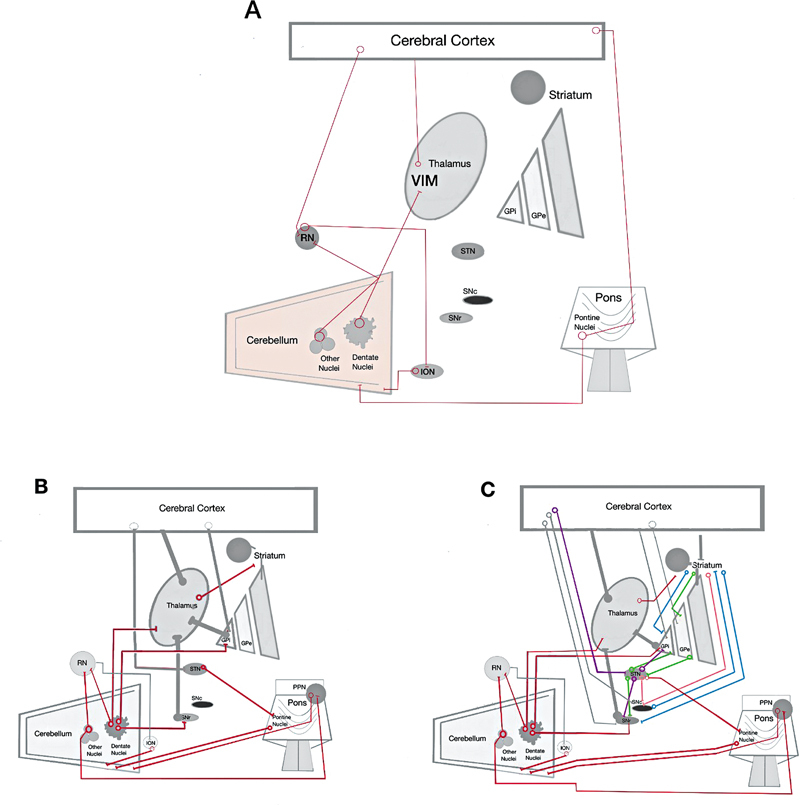
Abbreviations: ET, essential tremor; GPi, globus pallidus internus; GPe, globus pallidus externus; ION, inferior olivary nucleus; MLR, mesencephalic locomotor region; PPN, pedunculopontine nucleus; RN, red nucleus; SNc, substantia nigra pars compacta; SNr, substantia nigra pars reticulata; STN, subthalamic nucleus. Simplified diagrams illustrating the principal interrelationships among brain structures involved in essential tremor (ET) and other movement disorders. Connections belonging to various pathways are depicted in grey. ( **A** ) Cerebello-thalamo-cortical loop. ( **B** ) Main cerebellar connections associated with ET (red). ( **C** ) Superimposed representation of major motor pathways. Classic cortico -basal ganglia -thalamo -cortical circuits are shown as follows: direct pathway (blue), indirect pathway (green), and hyperdirect pathway (purple). Additional interconnections among the basal ganglia are illustrated in salmon, and the main cerebellar connections in red. **Adapted from:** Camargo et al. 34

Recent advancements in ET's genetics, environmental factors, animal models, pathology, and physiology emphasize the cerebellum's crucial role and connections within the cerebello-thalamo-cortical circuit ( Figure 1 ). These insights pinpoint significant therapeutic targets for ET and enhance our understanding of how cerebellar dysfunction contributes to the rhythmic, involuntary movements associated with this disorder. 1

### Cerebellum and pathophysiology of ET

There is no question that the cerebellum plays a crucial role in the pathways implicated in the pathophysiology of ET. What remains to be fully elucidated are the cellular and molecular dynamics within the cerebellum that disrupt normal movement modulation enough to contribute to tremor development. 1

Complex inputs from peripheral sources enter the cerebellum via the inferior cerebellar peduncle. These originate from the inferior olivary nucleus through climbing fibers, as well as from the vestibular nuclei, the accessory cuneate nucleus, and Clarke's dorsal nucleus via mossy fibers to the cerebellar cortex. Commands from the cerebral cortex reach the cerebellum through the middle cerebellar peduncle, consisting of mossy fibers from the pontine nuclei to the cerebellar cortex. The cerebellum's outputs are mediated by its deep nuclei (dentate, interposed, and fastigial), which receive projections from Purkinje cells.

These cells, the sole efferent neurons of the cerebellar cortex, are GABAergic and inhibitory. Furthermore, they are characterized by strong pacemaking activity, rigorously regulated by GABAergic neurotransmission from various interneurons, including stellate and basket cells. Basket cells exert inhibitory control over the soma of Purkinje cells, while stellate ones receive inputs from parallel fibers and provide inhibitory feedback to the dendrites of Purkinje cells.

**Figure 2:**
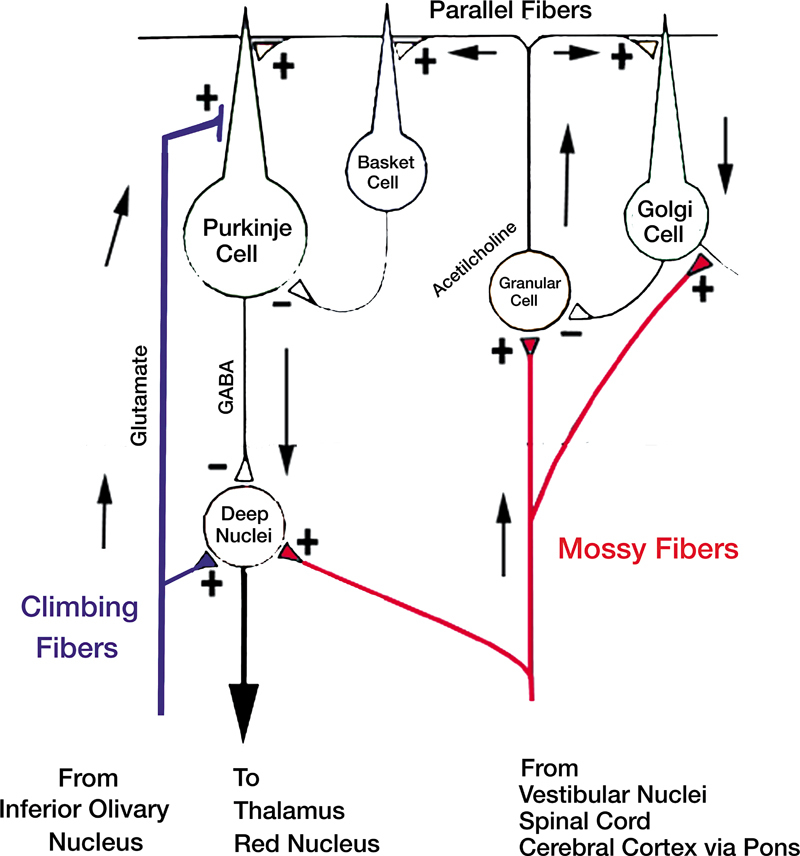
Simplified diagram illustrating the principal interrelationships among cerebellum cells in ET.

Golgi cells, also receiving inputs from parallel fibers, furnish inhibitory feedback to the granule cells that form the parallel fibers ( Figure 2 ). 12 13 14 Studies indicate that pathological changes in the cerebellum of patients with ET may occur in the cells comprising the input system, Purkinje, or even in those that modulate the system. 1 15 16 17 18

Regarding the efferent system, several pathological features centered around Purkinje cells have been identified in the cerebellum of patients with ET. These include a modest loss, swelling of axons known as torpedoes, displacement of cell bodies from their typical layer, and dendritic spine loss. 15 16 17 19 20 21

A study suggests that axonal swelling in Purkinje cells can enhance action potential fidelity, which may influence tremor dynamics. 22 While these morphological changes in the ET cerebellar cortex are evident, it raises the question of whether the downstream deep cerebellar nuclei are similarly affected. Research indicates no change in neuronal density within the dentate nucleus in this condition, suggesting that this region does not experience neuronal loss. 23 Furthermore, detailed postmortem analysis of GABA type A (GABA-A) and B (GABA-B) receptor binding in the dentate nucleus of ET patients shows a reduction in both, highlighting significant alterations in neurotransmitter receptor dynamics. 24

Numerous morphological changes in the ET cerebellum indicate degenerative alterations, primarily in Purkinje cells, although other associated cells also exhibit abnormalities. Notably, basket cells exhibit denser and more elongated plexuses surrounding the initial segment of Purkinje cell axons. 15 18 Furthermore, climbing fibers in ET form atypical synaptic connections with Purkinje cells within the parallel fiber synaptic territory. Among the various pathological features identified, the synaptic pathology of climbing fibers is particular to ET, suggesting a unique and distinctive mechanism underlying the disorder. 1

Compared to other degenerative cerebellar diseases such as spinocerebellar ataxias and multiple system atrophy, the cerebellum in ET exhibits an expansion of the climbing fiber synaptic territory, extending into the parallel fiber synaptic territory. In contrast, in ataxic cerebellums, there is a regression of climbing fiber synaptic territory. 15 25 26 This synaptic pathology of climbing fibers is consistently observed across ET patients with varying clinical characteristics, suggesting a direct correlation. In this condition, climbing fibers extend into the parallel fiber synaptic territory and form lateral connections to innervate multiple Purkinje cells. This specific pathological feature correlates with tremor severity. Originating from the inferior olive, which possesses intrinsic oscillatory properties, the extensive reach and multiple Purkinje cell innervation by climbing fibers could influence their pacemaking activity and alter cerebellar physiology, contributing to the manifestation of tremors. 1

Moreover, deficiencies in the synaptic pruning of climbing fibers to Purkinje cells, often linked to insufficient levels of the glutamate receptor δ2 subunit (GluRδ2) protein, lead to excessive cerebellar oscillatory activity. The protein acts as a master synaptic organizer, strictly regulating the territories innervated by climbing and parallel fibers on Purkinje cell dendrites, which may potentially suppress tremors. A deficiency in GluRδ2 can cause abnormal expansion of the climbing fiber synaptic territory in mice. Correspondingly, the cerebellum in ET shows reduced levels of this protein, which is associated with climbing fiber synaptic pathology. These findings suggest that GluRδ2-mediated climbing fiber synaptic pathology may be present in ET patients, impacting the development and severity of tremor symptoms. 1 27 28

Despite the logically sound and widely accepted hypothesis of the inferior olive nucleus's role as a pacemaker, modulating the action of Purkinje cells via climbing fibers glutamatergic action and potentially contributing to their degenerative process, it faces challenges in validation. 29 30

First, there is an apparent absence of functional and structural changes in the inferior olive nucleus in neuroimaging studies of patients with ET. The lack of morphological changes in postmortem examinations also supports this. 16 17 31

Secondly, while the inferior olive nucleus is undoubtedly capable of generating rhythmic burst activity, Purkinje cells, cerebellar nuclei, globus pallidus, thalamus, and sensorimotor cortex also possess this capability. These neurons with pacemaker properties are part of the cerebello-thalamo-cortical loop and other motor pathways, suggesting that the role of the inferior olive nucleus may be auxiliary within a more complex system. 29

Thirdly, animal models using harmaline or ibogaine may not be suitable models for simulating ET in humans. These work by inducing potentiation of the low-threshold voltage-gated calcium channels (CaV3.1) in the inferior olive nucleus crucial for the genesis of 4 to 10 Hz tremor-related rhythms. 29 32

### Cerebellum and brain circuits in ET patients

Given surface electromyography (EMG) and accelerometer findings point to a central origin of the tremor generator in ET, researchers have employed tremor frequency as a marker to investigate oscillatory activity and pinpoint the tremor source. Imaging techniques such as electroencephalogram (EEG) and magnetoencephalogram (MEG) have been used to assess functional connectivity and conduct frequency analysis in ET patients, revealing insights into the central mechanisms involved. 1 33 The major findings of these techniques indicate the role of the cerebello-thalamo-cortical loop in ET. 1

Recent studies, such as the one by Pan et al., 28 have demonstrated that enhanced cerebellar oscillatory activity can be directly recorded via cerebellar EEG in ET patients, underscoring the cerebellum's critical role. This activity is also significant in Parkinsonian tremors, as the cerebello-thalamo-cortical loop is vital for both conditions. 1 Interestingly, it has been suggested that the pathological mechanisms underlying ET and tremor in PD partially overlap. 1 34 Symptoms of both diseases appear simultaneously quite often, in up to 20% of cases. 35

The cerebellum's role in ET involves oscillatory activity that flows primarily towards the sensorimotor cortex, indicating a cerebellar origin of tremor oscillations. This is supported by findings of reduced cerebellocortical functional connectivity in ET, which correlates with tremor severity. 36 37

Schnitzler et al. 38 utilized MEG to identify a core structure consistently linked to the brainstem and cerebellar activities in ET, providing direct evidence of altered communication within a network that includes the cerebellum. The inferior olivary nucleus also plays a pivotal role by encoding tremor frequency. Research in both animal models and human patients indicates that the temporal coherence of neuronal firing in the olivocerebellum is closely linked to frequency-dependent cerebellar oscillations. Some EEG studies have shown that such oscillations persist even when tremor symptoms are suppressed by thalamic deep brain stimulation, suggesting that they do not depend on reciprocal interactions with the thalamus, highlighting the cerebellum's autonomous role in tremor generation. 39

Intrathalamic recordings indicate that the highest concentration of neurons with oscillatory characteristics is found within the ventral intermediate nucleus (VIM) of the thalamus, as noted by Hua et al. 40 This strong neuroanatomical link between the cerebellar nuclei and Vim neurons serves as indirect evidence of cerebellar pathology.

Buijink et al. 41 employed a combination of EMG and functional magnetic resonance imaging (fMRI) to simultaneously record the peripheral manifestations of tremor and the underlying brain activity. They reported that variations in tremor during a motor task exert an excitatory influence on both the extrinsic connectivity from cerebellar lobule V to the thalamus and the intrinsic activity within these regions. They also found that the motor network is notably compromised in ET, characterized by decreased connectivity between cortical and cerebellar motor regions during motor tasks, which correlates with an increase in clinical tremor severity. Furthermore, they noted that enhanced functional connectivity between right cerebellar lobules I to IV and the left thalamus is associated with more severe tremor symptoms. 41

Extending these findings, Contarino et al. 42 explored the neurological underpinnings of tremors using an EMG regressor. They established that voluntary movements activate the contralateral motor cortex, supplementary motor area, and ipsilateral cerebellum. Additionally, their analysis using a tremor frequency-tuned EMG regressor highlighted connections between tremor activity and activation in both the ipsilateral cerebellum and contralateral thalamus. Intriguingly, the specific sites of thalamic activation varied among patients and were not localized to the VIM, suggesting a complex, patient-specific pathophysiology underpinning tremors. 42

## INSIGHTS FROM GENETICS

The intricate role of genetics in the etiology of ET is underscored by the high prevalence of positive family history in patients, ranging from 20 to 90%, and the phenomenon of genetic anticipation, where tremor manifests at an earlier age in successive generations. 43 44 Genome-wide association studies (GWAS) have identified several genetic variants in genes such as *LINGO1* , *SLC1A2* , *STK32B* , *PPARGC1A* , and *CTNNA3* linked to ET, though these findings have yet to be replicated consistently. Additionally, exome studies have pinpointed genes associated with familial ET—such as *FUS* , *HTRA2* , *TENM4, FUS, SORT1, SCN11A, NOTCH2NLC, NOS3, KCNS2, HAPLN4, USP46, CACNA1G, SLIT3, CCDC183, MMP10* , and *GPR151* —although these appear to represent private polymorphisms unique to specific families, highlighting the need for further research to identify the genes responsible for ET. 44

Dysfunctions in the *LINGO1* gene may contribute to the loss of Purkinje cells and axonal damage, potentially leading to ET. Notably, the rs9652490 and rs11856808 variants within intron 3 of the *LINGO1* gene were identified as potential risk factors in the first GWAS on ET patients. 45 Furthermore, dysfunction in the *SLC1A2* gene, which regulates glutamate uptake, could lead to elevated levels, increasing the risk of neurotoxicity. This is particularly significant given that increased expression of *SLC1A2* has been observed in the inferior olive—a key area involved in generating the oscillations thought to underlie tremor. 46 47

Complementing genomic studies, transcriptomic research provides insights into differentially expressed genes (DEGs) that reflect changes in the molecular environment induced by the disease rather than the genetic mutations causing it. This approach bridges the gap between genomics and proteomics, offering a dynamic perspective on how genetic and environmental changes interact and contribute to the pathophysiology of ET. 48 For instance, a pioneering study by Liao et al. 49 utilized RNA sequencing to analyze postmortem tissue from the cerebellar cortex and dentate nucleus of ET patients, revealing significant transcriptomic variability that suggests different brain regions may uniquely contribute to the disorder's pathology.

Further, the study highlighted several underexpressed genes in the cerebellar cortex, such as *SHF* and *CACNA1A* . The latter is critical for the function of CaV2.1 voltage-gated calcium channels in Purkinje cells and is linked to familial hemiplegic migraine—a disorder that also features tremors. This suggests a possible mechanistic link between the dysregulation of calcium channels and tremor manifestation in ET. 49 Moreover, genes like *CACNA1A* and *CACNA1C* , which are involved in calcium channel functioning within the olivocerebellar circuitry, were found to be differentially expressed, aligning with findings from animal models that demonstrated enhanced neuronal synchrony and rhythmicity. 50 51

In 2023, Martuscello et al. 52 advanced this line of inquiry by focusing on the transcriptomic profiles of Purkinje cells—the cell type thought to degenerate in ET. Using laser capture microdissection, they analyzed the transcriptomes of such cells from ET patients and healthy controls, identifying key genes that differed in expression. This cell-type-specific analysis is critical for understanding the underlying mechanisms of the condition and could pave the way for targeted therapeutic interventions. This study not only reinforces the importance of the cerebellum in ET but also highlights the potential for transcriptomic approaches to uncover novel insights into the disease's molecular underpinnings. 52

## INSIGHTS FROM CLINICAL FINDINGS

In 2018, the International Parkinson and Movement Disorders Society (MDS) consensus standardized the ET definition and diagnostic criteria. These criteria include an isolated syndrome of bilateral upper limb action tremors with or without occurrences in other locations (such as the head, voice, or lower limbs), in the absence of other neurological signs such as dystonia, ataxia, or parkinsonism. This clinical entity must present a time course of at least 3 years, and all exclusion criteria must be absent ( Table 1 ). 8

**Table 1:** Diagnostic criteria of essential tremor and ET-plus 8

2018 MDS consensus statement on the classification of tremor diagnostic criteria for essential tremor
• Isolated tremor syndrome of bilateral upper limb action tremor. • At least 3 years of duration. • With or without tremor in other locations (e.g., the head, voice, or lower limbs). • Absence of other neurological signs, such as dystonia, ataxia, or parkinsonism.
Definition of ET-Plus: Tremor with the characteristics of ET and additional neurological signs of uncertain significance such as impaired tandem gait, questionable dystonic posturing, memory impairment, or other neurologic signs of unknown significance that do not suffice to make an additional syndrome classification or diagnosis. Cases of ET with tremor at rest should be classified here.
Exclusion criteria for ET and ET-Plus: • Isolated focal tremors (voice, head). • Orthostatic tremor with a frequency > 12 Hz. • Task- and position-specific tremors. • Sudden onset and step-wise deterioration.

Abbreviations: ET, essential tremor; MDS, Movement Disorders Society.

In the last two decades, several advances in clinical and pathophysiological characterization of ET challenged its definition as a homogeneous condition. In the 2018 MDS consensus, these studies were taken into consideration, and an essential tremor-plus (ET-Plus) definition was designed as a “placeholder” classification to group patients with prototypical findings associated with mild neurological signs of uncertain significance, insufficient to establish an alternative diagnosis. These neurological signs include motor symptoms, such as tandem gait ataxia and dystonia, and non-motor symptoms like cognitive impairment ( Table 1 ). 2 8 53 54 55 56 57 58 59

### ET-Plus and tandem gait ataxia/cerebellar findings

Since 1949, with the seminal paper by Critchley, 60 cerebellar findings have been described in ET. 61 Gait ataxia is one of its underestimated nontremulous features. A recent systematic review on the subject, including 23 studies, demonstrated an odds ratio of 7.03 for tandem gait ataxia compared to healthy controls and estimated that balance problems occurred in 42% of ET cases. It is also noteworthy that the qualitative gait analysis reported a pattern of impairment similar to the observed in cerebellar disorders, apart from a wide base of support, which was not shared among these pathologies. 62

A prospective longitudinal study in a population of 149 elderly patients with ET showed that those presenting with ataxia experienced gradual worsening of the gait disorder, with more missteps in examination, fewer seconds in tandem stance, and a cumulative number of falls and near-falls. This study, however, did not include age-matched healthy controls. 63

Other cerebellar findings, such as slower smooth pursuit, some forms of nystagmus, saccadic dysmetria, and reduced performance in predictive motor timing tasks, might be present in ET and are well documented. On the other hand, scanning or dysarthric speech, dysmetria, and dysdiadochokinesis are not expected and should prompt an alternative diagnosis. 53 64

Although the conception of the ET-Plus entity was an opportunity to establish the heterogeneity of clinical findings in the condition, it also raised several criticisms from experts worldwide. 53 54 55 56 57 One of the main points of criticism is the fact that several movement disorders, such as spinocerebellar ataxias, dystonia, and PD, present a high degree of clinical heterogeneity. Frequently, different clinical phenotypes exist within the same genotype, and these different presentations do not represent another entity but rather subsets of the same disease. This should be the case for ET, thus rendering the concept of another clinical entity named ET-Plus unnecessary. 53 54 55 56 57

## NEUROIMAGING INSIGHTS

Cerebellar abnormalities have been increasingly recognized in ET pathophysiology through neuroimaging studies. Current knowledge of the related cerebello-thalamo-cortical circuit dysfunction can be summarized in two theories. The first is an increased cerebellar oscillatory activity, and the second is cerebellar disconnection, or “decoupling”. 65 They are supported by different modalities of neuroimaging studies, which can be divided into gray and white matter structural abnormalities observed in diffusion tensor imaging (DTI) and voxel-based morphometry (VBM) analysis. Additionally, there are more advanced methods, including molecular imaging, which comprises single-photon emission computed tomography (SPECT), positron-emission tomography (PET), and MRI spectroscopy studies focusing on metabolic and neurotransmitter abnormalities. Lastly, functional imaging studies use fMRI to observe resting-state activated territories and EMG-fMRI changes during tasks. 65

### Structural abnormalities

Subtle and variable structural abnormalities of cerebellar gray and white matter are the most frequent findings in MRI studies. 66 However, there is significant heterogeneity in the results of structural imaging studies. Most have reported cerebellar mild gray matter (GM) atrophy in varying locations, including the vermis, lobules IV and V, and the posterior and anterior lobes. 66 67 68 69 70 71 In contrast, other studies reported no significant differences and even found subtle cortical abnormalities, including increased volume of the supplementary motor area. 9 10 72 Additionally, white matter (WM) changes may be seen in the cerebellar peduncles, right cerebellum, left medulla, right parietal lobe, and right limbic lobe, contributing to the cerebellar decoupling hypothesis. 73 74

Somatotopic organization of the cerebellum should be considered, as exemplified by a study of ET patients with hand and head tremor, which found abnormal vermis and lobule IV volume. Meanwhile, ET patients with only hand tremor had no significant difference compared to healthy controls. 67 71

### Molecular neuroimaging abnormalities

The MRI dpectroscopy studies have largely supported the role of ET as a neurodegenerative disorder. The main findings involve N-acetyl aspartate/choline (NAA/Cho) ratio reduction, reflecting neuronal loss, with one study reporting an inverse correlation with arm tremor severity. 75 76

Neurotransmitter abnormalities can be assessed with specific radioligands, with the most impactful being the ^11^ C-flumazenil PET. This radioligand is designed to reflect GABA-A receptor function, and one study demonstrated increased binding in the cerebellum (dentate nucleus), ventrolateral thalamus, and lateral premotor cortex. These findings suggest GABAergic dysfunction, though it remains unclear whether this is due to a reactive receptor upregulation or neuronal loss, but it is possibly related to overactivity in the tremor network. 77

### Functional abnormalities

The first functional studies utilized SPECT and PET to evaluate cerebral blood flow (CBF) or glucose metabolism, reflecting neuronal activation. Increased bilateral cerebellar CBF was found in ET patients, 78 79 mitigated by ethanol consumption. 80 One study revealed that PET imaging with 2-([18]F)fluoro-2-deoxy-D-glucose (FDG) hypermetabolism in the left thalamus and right cerebellar posterior lobe in 42 ET patients who underwent the Gamma Knife VIM procedure, which may be reversible after treatment. Additionally, there was a decrease in metabolic consumption in cortical areas, including the left temporal, bilateral middle, and inferior frontal gyri. Also, hypometabolism in the right temporo-occipital area, right retrosplenium, and posterior cingulate area, in addition to high connectivity between temporo-occipital and thalamic areas, were predictive of nonresponse to treatment. 81

Currently, most functional imaging studies use fMRI with blood oxygenation level-dependent (BOLD) sequences to assess neuronal activation and its association with motor symptoms during a resting state or a postural/kinetic task. Several fMRI studies have shown abnormal activity in the cerebellar hemispheres. There has been association between ET and hyper- and hypoactivity in numerous cerebellar segments, with the most consistent involvement in lobules IV to VI and increased activity in the contralateral sensorimotor cortex. Resting-state fMRI has more frequently reported hypoactivity, while studies during action and postural tasks have more commonly shown cerebellar hyperactivity. 10 36 41 42 82 83 84 85 86 87 88 89 90 91 However, some ET patients may have decreased dentate nucleus connectivity with the SMA, the primary sensorimotor cortex, and the prefrontal cortex, showing an inverse correlation with tremor severity and disease duration. 92 Hence, in some cases, there may be an excess of activity in central oscillators in the cerebello-thalamo-cortical network, while, in others it may be related to a functional disconnection of cerebellar output. 65 66 93 94 95

The functional abnormalities in ET patients can be modulated with deep brain stimulation (DBS) treatment, as shown in a study with 16 participants, where fMRI abnormalities were compared with ON/OFF DBS caudal zona incerta. The postural tremor was associated with increased activity in the contralateral primary sensorimotor and premotor cortices, SMA, thalamus, and bilateral cerebellum (right lobules IV, V, VI, vermis, VIII, and left VI). When the treatment began, the activity decreased in the primary sensorimotor cortex and cerebellar lobule VIII during postural tasks. In contrast, resting tremors improved with this treatment, which was related to the increased activity in the SMA and cerebellar lobule V. 96

## TREATMENT INSIGHTS

### Pharmacological treatments

The clinical pharmacology of ET strongly implicates cerebellar involvement, mainly through alterations in GABAergic transmission. Commonly prescribed medications for ET, such as primidone, phenobarbital, benzodiazepines, gabapentin, and topiramate, all work primarily by enhancing this transmission, underscoring the hypothesis of reduced GABAergic tone in ET. 97 The central role of Purkinje cells, the primary GABAergic output of the cerebellum, suggests that a reduction in the cells' functionality could lead to decreased GABAergic tone, contributing to the pathology. 7

Research has mainly focused on the structure and functionality of GABA-A receptors, which are predominantly composed of 2 α (6 types), 2 β (3 types), and 1 δ or γ subunit. Genetic and pharmacological studies underline the importance of these receptors in managing ET symptoms. For instance, GABA-A receptor α1 knockout mice display postural and kinetic tremors, emphasizing the critical role of specific receptor subunits in the symptoms. 98 Further, positive allosteric modulators of the α6 subunit of GABA-A receptors have been shown to improve tremor in animal models. 99

The SAGE-324/BIIB124 is a neuroactive steroid-positive allosteric modulator of GABA-A receptors with a particular affinity for α4β3δ subtypes. In a recent phase-2 study involving 67 ET patients (NCT04305275), this modulator demonstrated a significant decrease in tremor severity. Despite these encouraging results, the trial also reported significant adverse effects: a 62% rate of dose reductions and a 38% discontinuation rate among participants, underscoring the challenges in balancing efficacy with tolerability. 100

The use of anticholinergic agents to treat tremor dates back to Charcot and Gowers. 4 The traditional understanding has been that anticholinergics alleviate tremors by correcting a striatal neurotransmitter imbalance, characterized by reduced dopaminergic and increased cholinergic activity. 101 However, recent studies have expanded this view, suggesting that the anticholinergic effects on tremors may also involve modulation of cerebellar circuits. 102 103

Acetylcholine (ACh) plays a critical role in cerebellar function, with dense cholinergic projections terminating in the granule cell layer. These anatomical findings suggest that ACh significantly influences cerebellar processing and associated behaviors.

Fore et al. 104 demonstrated in vitro that ACh exerts a prolonged inhibitory effect on Golgi cells via muscarinic receptor activation, leading to reduced synaptic inhibition onto granule cells. Additionally, muscarinic receptor activation on mossy fibers diminishes the excitatory input to granule cells. This concurrent reduction in excitation and inhibition alters spike probability in a heterogeneous manner, enhancing excitability in some granule cells while suppressing it in others. Notably, ACh preferentially increases the excitability of granule cells that are strongly inhibited, supporting the idea that this mechanism is stimulus-specific and may be essential for cerebellar learning. These findings imply that cholinergic neuromodulation could selectively enhance learning for specific mossy fiber inputs, depending on behavioral context or stimulus salience. Thus, ACh may play a key role in tuning cerebellar plasticity and modulating the gain of cerebellar signal processing. Nevertheless, the *in vivo* mechanisms regulating its release and precise impact on synaptic and network dynamics have yet to be fully elucidated. 104

### Neurosurgical treatment

The cerebellum exerts significant influence through glutamatergic inputs to the VIM nucleus via deep cerebellar nuclei (dentate, interposed, and fastigial). 105 This connection is fundamental to the efficacy of DBS targeting the VIM and its afferent fibers, the dentato-rubro-thalamic tract (DRRT), particularly in alleviating tremor across various disorders, including ET. 106

The VIM nucleus is a principal target for DBS and thalamotomy aimed at tremor management. The DRTT fibers from the cerebellum traverse the brachium conjunctivum, pass anterior to the red nucleus, and ascend into the VIM. The latter then projects to the ipsilateral motor cortex (M1) and associated cortical areas such as the premotor cortex, supplementary motor area, and presupplementary motor area. 106

Clinical outcomes from VIM stimulation are highly significant for tremor reduction, particularly in ET. A thorough review of 40 studies reported that unilateral VIM DBS resulted in a tremor reduction of 53.4 to 62.8% in this cohort after 12 months, with action tremors showing the best response, with improvements up to 78.9%. Additionally, bilateral stimulation has been shown to be both safe and effective, providing greater tremor relief (range: 66–78%) and better management of axial and voice tremors. 107

In conclusion, the existing body of scientific literature emphasizes the intricate interactions among cerebellar dysfunction, disrupted neuronal signaling, and compromised feedback mechanisms that contribute to the pathophysiology of ET. These insights affirm the central role of the cerebellum in this condition, mainly through abnormal oscillations and impaired cerebellocortical connectivity. This understanding supports the notion that therapies targeting cerebellar activity could effectively mitigate tremor symptoms.

To date, a combination of genetic studies, neuropathological examinations, neurophysiological assessments, and various neuroimaging techniques have demonstrated functional, neurotransmitter-related, and structural abnormalities within the cerebello-thalamo-cortical circuit. These findings collectively suggest ET as a neurodegenerative syndrome with diverse etiologies and clinical manifestations.

However, caution must be exercised in interpreting these findings due to the limitations of most studies, which typically involve small sample sizes and are restricted to cross-sectional analyses. These limitations hinder our ability to draw definitive conclusions about causality and the precise relationship between the cerebellum and ET. Ongoing and future longitudinal studies are essential to provide a more robust understanding of these associations and to confirm the cerebellum's definitive role in this condition.

## References

[1] Pan M K Kuo S H Essential tremor: Clinical perspectives and pathophysiology J Neurol Sci 2022 435 120198 10.1016/j.jns.2022.120198 35299120 PMC10363990

[2] Elble R J The essential tremor syndromes Curr Opin Neurol 2016 29 04 507 512 10.1097/WCO.0000000000000347 27257943

[3] Charcot J-M De la paralysie agitante Paris Bureaux du Progrés Médical 1872. T. 1, p. 155–188

[4] Goetz C G The history of Parkinson's disease: early clinical descriptions and neurological therapies Cold Spring Harb Perspect Med 2011 1 01 a008862 10.1101/cshperspect.a008862 22229124 PMC3234454

[5] Teive H A [Charcot's contribution to Parkinson's disease] Arq Neuro-Psiquiatr 1998 56 01 141 145 10.1590/s0004-282X1998000100026 9686138

[6] Louis E D Broussolle E Goetz C G Krack P Kaufmann P Mazzoni P Historical underpinnings of the term essential tremor in the late 19th century Neurology 2008 71 11 856 859 10.1212/01.wnl.0000325564.38165.d1 18779514 PMC3461999

[7] Louis E D Essential Tremor: A Common Disorder of Purkinje Neurons? Neuroscientist 2016 22 02 108 118 10.1177/1073858415590351 26044399 PMC5467972

[8] Tremor Task Force of the International Parkinson and Movement Disorder Society Bhatia K P Bain P Bajaj N Elble R J Hallett M Louis E D Consensus Statement on the classification of tremors. from the task force on tremor of the International Parkinson and Movement Disorder Society Mov Disord 2018 33 01 75 87 10.1002/mds.27121 29193359 PMC6530552

[9] Gallea C Popa T Garcia-Lorenzo D Valabregue R Legrand A-P Marais L Intrinsic signature of essential tremor in the cerebello-frontal network Brain 2015 138(Pt 10):2920 2933 10.1093/brain/awv171 26115677 PMC4747645

[10] Nicoletti V Cecchi P Pesaresi I Frosini D Cosottini M Ceravolo R Cerebello-thalamo-cortical network is intrinsically altered in essential tremor: evidence from a resting state functional MRI study Sci Rep 2020 10 01 16661 10.1038/s41598-020-73714-9 33028912 PMC7541442

[11] Zhang X Santaniello S Role of cerebellar GABAergic dysfunctions in the origins of essential tremor Proc Natl Acad Sci U S A 2019 116 27 13592 13601 10.1073/pnas.1817689116 31209041 PMC6612915

[12] Apps R Garwicz M Anatomical and physiological foundations of cerebellar information processing Nat Rev Neurosci 2005 6 04 297 311 10.1038/nrn1646 15803161

[13] Suzuki L Coulon P Sabel-Goedknegt E H Ruigrok T JH Organization of cerebral projections to identified cerebellar zones in the posterior cerebellum of the rat J Neurosci 2012 32 32 10854 10869 10.1523/JNEUROSCI.0857-12.2012 22875920 PMC6621006

[14] Fanning A Kuo S H Clinical Heterogeneity of Essential Tremor: Understanding Neural Substrates of Action Tremor Subtypes Cerebellum 2024 23 06 2497 2510 10.1007/s12311-023-01551-3 37022657 PMC10556200

[15] Kuo S H Erickson-Davis C Gillman A Faust P L Vonsattel J PG Louis E D Increased number of heterotopic Purkinje cells in essential tremor J Neurol Neurosurg Psychiatry 2011 82 09 1038 1040 10.1136/jnnp.2010.213330 20802031 PMC3856652

[16] Louis E D Faust P L Vonsattel J PG Honig L S Rajput A Robinson C A Neuropathological changes in essential tremor: 33 cases compared with 21 controls Brain 2007 130(Pt 12):3297 3307 10.1093/brain/awm266 18025031

[17] Louis E D Faust P L Essential tremor pathology: neurodegeneration and reorganization of neuronal connections Nat Rev Neurol 2020 16 02 69 83 10.1038/s41582-019-0302-1 31959938

[18] Erickson-Davis C R Faust P L Vonsattel J P Gupta S Honig L S Louis E D “Hairy baskets” associated with degenerative Purkinje cell changes in essential tremor J Neuropathol Exp Neurol 2010 69 03 262 271 10.1097/NEN.0b013e3181d1ad04 20142764 PMC2865233

[19] Babij R Lee M Cortés E Vonsattel J P Faust P L Louis E D Purkinje cell axonal anatomy: quantifying morphometric changes in essential tremor versus control brains Brain 2013 136(Pt 10):3051 3061 10.1093/brain/awt238 24030953 PMC3784286

[20] Yu M Ma K Faust P L Honig L S Cortés E Vonsattel J PG Louis E D Increased number of Purkinje cell dendritic swellings in essential tremor Eur J Neurol 2012 19 04 625 630 10.1111/j.1468-1331.2011.03598.x 22136494 PMC3297734

[21] Louis E D Lee M Babij R Ma K Cortés E Vonsattel J PG Faust P L Reduced Purkinje cell dendritic arborization and loss of dendritic spines in essential tremor Brain 2014 137(Pt 12):3142 3148 10.1093/brain/awu314 25367027 PMC4240305

[22] Lang-Ouellette D Gruver K M Smith-Dijak A Blot F GC Stewart C A de Blavous PdV Purkinje cell axonal swellings enhance action potential fidelity and cerebellar function Nat Commun 2021 12 01 4129 10.1038/s41467-021-24390-4 34226561 PMC8257784

[23] Louis E D Hernández N Dyke J P Ma R E Dydak U In vivo dentate nucleus gamma-aminobutyric acid concentration in essential tremor vs. controls Cerebellum 2018 17 02 165 172 10.1007/s12311-017-0891-4 29039117 PMC5851820

[24] Paris-Robidas S Brochu E Sintes M Emond V Bousquet M Vandal M Defective dentate nucleus GABA receptors in essential tremor Brain 2012 135(Pt 1):105 116 10.1093/brain/awr301 22120148

[25] Louis E D Kerridge C A Chatterjee D Martuscello R T Diaz D T Koeppen A H Contextualizing the pathology in the essential tremor cerebellar cortex: a patholog-omics approach Acta Neuropathol 2019 138 05 859 876 10.1007/s00401-019-02043-7 31317229 PMC7285399

[26] Louis E D Martuscello R T Gionco J T Hartstone W G Musacchio J B Portenti M Histopathology of the cerebellar cortex in essential tremor and other neurodegenerative motor disorders: comparative analysis of 320 brains Acta Neuropathol 2023 145 03 265 283 10.1007/s00401-022-02535-z 36607423 PMC10461794

[27] Miyazaki T Yamasaki M Takeuchi T Sakimura K Mishina M Watanabe M Ablation of glutamate receptor GluRδ2 in adult Purkinje cells causes multiple innervation of climbing fibers by inducing aberrant invasion to parallel fiber innervation territory J Neurosci 2010 30 45 15196 15209 10.1523/JNEUROSCI.0934-10.2010 21068325 PMC6633829

[28] Pan M K Li Y S Wong S B Ni C L Wang Y M Liu W C Cerebellar oscillations driven by synaptic pruning deficits of cerebellar climbing fibers contribute to tremor pathophysiology Sci Transl Med 2020 12 526 eaay1769 10.1126/scitranslmed.aay1769 31941824 PMC7339589

[29] Louis E D Lenka A The Olivary Hypothesis of Essential Tremor: Time to Lay this Model to Rest? Tremor Other Hyperkinet Mov (N Y) 2017 7 473 10.7916/D8FF40RX 28966877 PMC5618117

[30] Kosmowska B Wardas J The Pathophysiology and Treatment of Essential Tremor: The Role of Adenosine and Dopamine Receptors in Animal Models Biomolecules 2021 11 12 1813 10.3390/biom11121813 34944457 PMC8698799

[31] Louis E D Babij R Cortés E Vonsattel J-PG Faust P L The inferior olivary nucleus: a postmortem study of essential tremor cases versus controls Mov Disord 2013 28 06 779 786 10.1002/mds.25400 23483605 PMC3688649

[32] Cheng M M Tang G Kuo S H Harmaline-induced tremor in mice: videotape documentation and open questions about the model Tremor Other Hyperkinet Mov (N Y) 2013 3 tre-03-205-4668-1 10.7916/D8H993W3 PMC3863988 24386609

[33] Filip P Lungu O V Manto M U Bareš M Linking Essential Tremor to the Cerebellum: Physiological Evidence Cerebellum 2016 15 06 774 780 10.1007/s12311-015-0740-2 26530223

[34] Camargo C HF Ferreira-Peruzzo S A Ribas D IR Franklin G L Teive H AG Imbalance and gait impairment in Parkinson's disease: discussing postural instability and ataxia Neurol Sci 2024 45 04 1377 1388 10.1007/s10072-023-07205-w 37985635

[35] Froehner G Camargo C Fabiani G Meira A T Martins Filho R Munhoz R P Teive H AG Parkinson's Disease in Patients with Essential Tremor: A Prospective Clinical and Functional Neuroimaging Assessment Open Neurol J 2022 16 e1874205 X2202071 10.2174/1874205X-v16-e2202071

[36] Neely K A Kurani A S Shukla P Planetta P J Shukla A W Goldman J G Functional brain activity relates to 0–3 and 3–8 Hz force oscillations in essential tremor Cereb Cortex 2015 25 11 4191 4202 10.1093/cercor/bhu142 24962992 PMC4816778

[37] Muthuraman M Hellriegel H Paschen S Hofschulte F Reese R Volkmann J The central oscillatory network of orthostatic tremor Mov Disord 2013 28 10 1424 1430 10.1002/mds.25616 23926026

[38] Schnitzler A Münks C Butz M Timmermann L Gross J Synchronized brain network associated with essential tremor as revealed by magnetoencephalography Mov Disord 2009 24 11 1629 1635 10.1002/mds.22633 19514010

[39] Wang Y M Liu C W Chen S Y Lu L Y Liu W C Wang J H Neuronal population activity in the olivocerebellum encodes the frequency of essential tremor in mice and patients Sci Transl Med 2024 16 747 eadl1408 10.1126/scitranslmed.adl1408 38748772

[40] Hua S E Lenz F A Posture-related oscillations in human cerebellar thalamus in essential tremor are enabled by voluntary motor circuits J Neurophysiol 2005 93 01 117 127 10.1152/jn.00527.2004 15317839

[41] Buijink A WG Van der Stouwe A MM Broersma M Sharifi S Groot P FC Speelman J D Motor network disruption in essential tremor: a functional and effective connectivity study Brain 2015 138(Pt 10):2934 2947 10.1093/brain/awv225 26248468

[42] Contarino M F Groot P FC Van der Meer J N Bour L J Speelman J D Nederveen A J Is there a role for combined EMG-fMRI in exploring the pathophysiology of essential tremor and improving functional neurosurgery? PLoS One 2012 7 10 e46234 10.1371/journal.pone.0046234 23049695 PMC3462183

[43] Hopfner F Haubenberger D Galpern W R Gwinn K Van't Veer A White S Knowledge gaps and research recommendations for essential tremor Parkinsonism Relat Disord 2016 33 27 35 10.1016/j.parkreldis.2016.10.002 27769649 PMC5271603

[44] Jiménez-Jiménez F J Alonso-Navarro H García-Martín E Agúndez J AG Sleep disorders in essential tremor: systematic review and meta-analysis Sleep 2020 43 09 zsaa039 10.1093/sleep/zsaa039 32163585

[45] Stefansson H Steinberg S Petursson H Gustafsson O Gudjonsdottir I H Jonsdottir G A Variant in the sequence of the LINGO1 gene confers risk of essential tremor Nat Genet 2009 41 03 277 279 10.1038/ng.299 19182806 PMC3740956

[46] Thier S Lorenz D Nothnagel M Poremba C Papengut F Appenzeller S Polymorphisms in the glial glutamate transporter SLC1A2 are associated with essential tremor Neurology 2012 79 03 243 248 10.1212/WNL.0b013e31825fdeed 22764253 PMC3398434

[47] Siokas V Aloizou A M Tsouris Z Liampas I Aslanidou P Dastamani M Genetic Risk Factors for Essential Tremor: A Review Tremor Other Hyperkinet Mov (N Y) 2020 10 01 4 10.5334/tohm.67 32775018 PMC7394223

[48] Aboasali F Castonguay C E Medeiros M Dion P A Rouleau G A Tremor in the Age of Omics: An Overview of the Transcriptomic Landscape of Essential Tremor Cerebellum 2025 24 02 35 10.1007/s12311-025-01793-3 39853640

[49] Liao C Sarayloo F Rochefort D Houle G Akçimen F He Q Multiomics analyses identify genes and pathways relevant to essential tremor Mov Disord 2020 35 07 1153 1162 10.1002/mds.28031 32249994

[50] Mori Y Friedrich T Kim M S Mikami A Nakai J Ruth P Primary structure and functional expression from complementary DNA of a brain calcium channel Nature 1991 350(6317):398 402 10.1038/350398a0 1849233

[51] Miwa H Rodent models of tremor Cerebellum 2007 6 01 66 72 10.1080/14734220601016080 17366267

[52] Martuscello R T Sivaprakasam K Hartstone W Kuo S H Konopka G Louis E D Faust P L Gene Expression Analysis of Laser-Captured Purkinje Cells in the Essential Tremor Cerebellum Cerebellum 2023 22 06 1166 1181 10.1007/s12311-022-01483-4 36242761 PMC10359949

[53] Louis E D Essential tremor then and now: How views of the most common tremor diathesis have changed over time Parkinsonism Relat Disord 2018 46 01 S70 S74 10.1016/j.parkreldis.2017.07.010 28747278 PMC5696041

[54] Louis E D The evolving definition of essential tremor: What are we dealing with? Parkinsonism Relat Disord 2018 46 01 S87 S91 10.1016/j.parkreldis.2017.07.004 28747280 PMC5696078

[55] Louis E D The Essential Tremors: Evolving concepts of a family of diseases Front Neurol 2021 12 650601 10.3389/fneur.2021.650601 33841316 PMC8032967

[56] Louis E D “Essential Tremor Plus”: A Problematic Concept: Implications for Clinical and Epidemiological Studies of Essential Tremor Neuroepidemiology 2020 54 02 180 184 10.1159/000502862 32023613

[57] Louis E D Bares M Benito-Leon J Fahn S Frucht S J Jankovic J Essential tremor-plus: a controversial new concept Lancet Neurol 2020 19 03 266 270 10.1016/S1474-4422(19)30398-9 31767343 PMC10686582

[58] Hopfner F Deuschl G Is essential tremor a single entity? Eur J Neurol 2018 25 01 71 82 10.1111/ene.13454 28905504

[59] Vidailhet M Essential tremor-plus: a temporary label Lancet Neurol 2020 19 03 202 203 10.1016/S1474-4422(19)30442-9 31767342

[60] Critchley M Observations on essential (heredofamial) tremor Brain 1949 72(Pt. 2):113 139 10.1093/brain/72.2.113 18136705

[61] Benito-León J Labiano-Fontcuberta A Linking essential tremor to the cerebellum: Clinical evidence Cerebellum 2016 15 03 253 262 10.1007/s12311-015-0741-1 26521074

[62] Rao A K Louis E D Ataxic gait in Essential tremor: A disease associated feature? Tremor Other Hyperkinet Mov (N Y) 2019 9 10.7916/d8-28jq-8t52 PMC6691745 31413894

[63] Dowd H Zdrodowska M A Radler K H Cersonsky T EK Rao A K Huey E D Prospective longitudinal study of gait and balance in a cohort of elderly essential tremor patients Front Neurol 2020 11 581703 10.3389/fneur.2020.581703 33304305 PMC7691661

[64] Visser F Bour L J Lee Y X Brinke T RT Van Rootselaar A F Eye movement abnormalities in essential tremor versus tremor dominant Parkinson's disease Clin Neurophysiol 2019 130 05 683 691 10.1016/j.clinph.2019.01.026 30875535

[65] Van der Stouwe A MM Nieuwhof F Helmich R C Tremor pathophysiology: lessons from neuroimaging Curr Opin Neurol 2020 33 04 474 481 10.1097/WCO.0000000000000829 32657888

[66] Cerasa A Quattrone A Linking Essential Tremor to the Cerebellum-Neuroimaging Evidence Cerebellum 2016 15 03 263 275 10.1007/s12311-015-0739-8 26626626

[67] Cerasa A Messina D Nicoletti G Novellino F Lanza P Condino F Cerebellar atrophy in essential tremor using an automated segmentation method AJNR Am J Neuroradiol 2009 30 06 1240 1243 10.3174/ajnr.A1544 19342539 PMC7051361

[68] Bagepally B S Bhatt M D Chandran V Saini J Bharath R D Vasudev M K Decrease in cerebral and cerebellar gray matter in essential tremor: a voxel-based morphometric analysis under 3T MRI J Neuroimaging 2012 22 03 275 278 10.1111/j.1552-6569.2011.00598.x 21447032

[69] Bhalsing K S Upadhyay N Kumar K J Saini J Yadav R Gupta A K Pal P K Association between cortical volume loss and cognitive impairments in essential tremor Eur J Neurol 2014 21 06 874 883 10.1111/ene.12399 24612409

[70] Lin C H Chen C M Lu M K Tsai C H Chiou J C Liao J R Duann J R VBM Reveals Brain Volume differences between Parkinson's disease and essential tremor patients Front Hum Neurosci 2013 7 247 10.3389/fnhum.2013.00247 23785322 PMC3682128

[71] Quattrone A Cerasa A Messina D Nicoletti G Hagberg G E Lemieux L Essential head tremor is associated with cerebellar vermis atrophy: a volumetric and voxel-based morphometry MR imaging study AJNR Am J Neuroradiol 2008 29 09 1692 1697 10.3174/ajnr.A1190 18653686 PMC8118768

[72] Daniels C Peller M Wolff S Alfke K Witt K Gaser C Voxel-based morphometry shows no decreases in cerebellar gray matter volume in essential tremor Neurology 2006 67 08 1452 1456 10.1212/01.wnl.0000240130.94408.99 17060572

[73] Benito-León J Alvarez-Linera J Hernández-Tamames J A Alonso-Navarro H Jiménez-Jiménez F J Louis E D Brain structural changes in essential tremor: voxel-based morphometry at 3-Tesla J Neurol Sci 2009 287(1-2):138 142 10.1016/j.jns.2009.08.037 19717167

[74] Juttukonda M R Franco G Englot D J Lin Y C Petersen K J Trujillo P White matter differences between essential tremor and Parkinson disease Neurology 2019 92 01 e30 e39 10.1212/WNL.0000000000006694 30504432 PMC6336163

[75] Louis E D Shungu D C Chan S Mao X Jurewicz E C Watner D Metabolic abnormality in the cerebellum in patients with essential tremor: a proton magnetic resonance spectroscopic imaging study Neurosci Lett 2002 333 01 17 20 10.1016/s0304-3940(02)00966-7 12401550

[76] Pagan F L Butman J A Dambrosia J M Hallett M Evaluation of essential tremor with multi-voxel magnetic resonance spectroscopy Neurology 2003 60 08 1344 1347 10.1212/01.wnl.0000065885.15875.0d 12707440

[77] Boecker H Weindl A Brooks D J Ceballos-Baumann A O Liedtke C Miederer M GABAergic dysfunction in essential tremor: an 11C-flumazenil PET study J Nucl Med 2010 51 07 1030 1035 10.2967/jnumed.109.074120 20554735

[78] Colebatch J G Findley L J Frackowiak R SJ Marsden C D Brooks D J Preliminary report: activation of the cerebellum in essential tremor Lancet 1990 336(8722):1028 1030 10.1016/0140-6736(90)92489-5 1977019

[79] Wills A J Jenkins I H Thompson P D Findley L J Brooks D J Red nuclear and cerebellar but no olivary activation associated with essential tremor: a positron emission tomographic study Ann Neurol 1994 36 04 636 642 10.1002/ana.410360413 7944296

[80] Boecker H Wills A J Ceballos-Baumann A Samuel M Thompson P D Findley L J Brooks D J The effect of ethanol on alcohol-responsive essential tremor: a positron emission tomography study Ann Neurol 1996 39 05 650 658 10.1002/ana.410390515 8619551

[81] Verger A Witjas T Carron R Eusebio A Boutin E Azulay J P Metabolic Positron Emission Tomography Response to Gamma Knife of the Ventral Intermediate Nucleus in Essential Tremor Neurosurgery 2019 84 06 E294 E303 10.1093/neuros/nyy340 30085092

[82] Louis E D Huang C C Dyke J P Long Z Dydak U Neuroimaging studies of essential tremor: how well do these studies support/refute the neurodegenerative hypothesis? Tremor Other Hyperkinet Mov (N Y) 2014 4 235 10.7916/D8DF6PB8 24918024 PMC4038743

[83] Broersma M Van der Stouwe A MM Buijink A WG De Jong B M Groot P FC Speelman J D Bilateral cerebellar activation in unilaterally challenged essential tremor Neuroimage Clin 2015 11 1 9 10.1016/j.nicl.2015.12.011 26909321 PMC4732188

[84] Fang W Chen H Wang H Zhang H Puneet M Liu M Essential tremor is associated with disruption of functional connectivity in the ventral intermediate Nucleus–Motor Cortex–Cerebellum circuit Hum Brain Mapp 2016 37 01 165 178 10.1002/hbm.23024 26467643 PMC6867464

[85] Holtbernd F Shah N J Imaging the Pathophysiology of Essential Tremor-A Systematic Review Front Neurol 2021 12 680254 10.3389/fneur.2021.680254 34220687 PMC8244929

[86] Bucher S F Seelos K C Dodel R C Reiser M Oertel W H Activation Mapping in Essential Tremor with Functional Magnetic Resonance lmaging Ann Neurol 1997 41 01 32 40 10.1002/ana.410410108 9005863

[87] Buijink A WG Broersma M Van der Stouwe A MM Van Wingen G A Groot P FC Speelman J D Maurits N M Van Rootselaar A F Rhythmic finger tapping reveals cerebellar dysfunction in essential tremor Parkinsonism Relat Disord 2015 21 04 383 388 10.1016/j.parkreldis.2015.02.003 25703340

[88] Mueller K Jech R Hoskovcová M Ulmanová O Urgošík D Vymazal J Růžička E General and selective brain connectivity alterations in essential tremor: A resting state fMRI study Neuroimage Clin 2017 16 468 476 10.1016/j.nicl.2017.06.004 28913163 PMC5587870

[89] Lenka A Bhalsing K S Panda R Jhunjhunwala K Naduthota R M Saini J Role of altered cerebello-thalamo-cortical network in the neurobiology of essential tremor Neuroradiology 2017 59 02 157 168 10.1007/s00234-016-1771-1 28062908

[90] Wang L Lei D Suo X Li N Lu Z Li J Resting-state fMRI study on drug-naive patients of essential tremor with and without head tremor Sci Rep 2018 8 01 10580 10.1038/s41598-018-28778-z 30002390 PMC6043592

[91] Yin W Lin W Li W Qian S Mou X Resting State fMRI Demonstrates a Disturbance of the Cerebello-Cortical Circuit in Essential Tremor Brain Topogr 2016 29 03 412 418 10.1007/s10548-016-0474-6 26868003

[92] Tikoo S Pietracupa S Tommasin S Bologna M Petsas N Bharti K Functional disconnection of the dentate nucleus in essential tremor J Neurol 2020 267 05 1358 1367 10.1007/s00415-020-09711-9 31974808

[93] Van den Berg K RE Helmich R C The role of the cerebellum in tremor – evidence from neuroimaging Tremor Other Hyperkinet Mov (N Y) 2021 11 49 10.5334/tohm.660 34820148 PMC8603856

[94] Helmich R C Toni I Deuschl G Bloem B R The pathophysiology of essential tremor and Parkinson's tremor Curr Neurol Neurosci Rep 2013 13 09 378 10.1007/s11910-013-0378-8 23893097

[95] Nieuwhof F Panyakaew P Van de Warrenburg B P Gallea C Helmich R C The patchy tremor landscape: recent advances in pathophysiology Curr Opin Neurol 2018 31 04 455 461 10.1097/WCO.0000000000000582 29750732

[96] Awad A Blomstedt P Westling G Eriksson J Deep brain stimulation in the caudal zona incerta modulates the sensorimotor cerebello-cerebral circuit in essential tremor Neuroimage 2020 209 116511 10.1016/j.neuroimage.2019.116511 31901420

[97] Rincon F Louis E D Benefits and risks of pharmacological and surgical treatments for essential tremor: disease mechanisms and current management Expert Opin Drug Saf 2005 4 05 899 913 10.1517/14740338.4.5.899 16111452

[98] Kralic J E Criswell H E Osterman J L O'Buckley T K Wilkie M E Matthews D B Genetic essential tremor in gamma-aminobutyric acidA receptor alpha1 subunit knockout mice J Clin Invest 2005 115 03 774 779 10.1172/JCI23625 15765150 PMC1052003

[99] Huang Y H Lee M T Hsueh H Y Knutson D E Cook J Mihovilovic M D Cerebellar α6GABA(A) receptors as a therapeutic target for essential tremor: proof-of-concept study with ethanol and pyrazoloquinolinones Neurotherapeutics 2023 20 02 399 418 10.1007/s13311-023-01342-y 36696034 PMC10121996

[100] Elble R J Ondo W G Lyons K E Qin M Garafola S Hersh B A Randomized Phase 2 KINETIC Trial Evaluating SAGE-324/BIIB124 in Individuals with Essential Tremor Mov Disord 2024 39 04 733 738 10.1002/mds.29731 38357797

[101] Duvoisin R C Cholinergic-anticholinergic antagonism in parkinsonism Arch Neurol 1967 17 02 124 136 10.1001/archneur.1967.00470260014002 4382112

[102] Bohnen N I Kanel P Koeppe R A Sanchez-Catasus C A Frey K A Scott P Regional cerebral cholinergic nerve terminal integrity and cardinal motor features in Parkinson's disease Brain Commun 2021 3 02 fcab109 10.1093/braincomms/fcab109 34704022 PMC8196256

[103] Jaarsma D Ruigrok T J Caffé R Cozzari C Levey A I Mugnaini E Voogd J Cholinergic innervation and receptors in the cerebellum Prog Brain Res 1997 114 67 96 10.1016/s0079-6123(08)63359-2 9193139

[104] Fore T R Taylor B N Brunel N Hull C Acetylcholine Modulates Cerebellar Granule Cell Spiking by Regulating the Balance of Synaptic Excitation and Inhibition J Neurosci 2020 40 14 2882 2894 10.1523/JNEUROSCI.2148-19.2020 32111698 PMC7117893

[105] Houk J C Wise S P Distributed modular architectures linking basal ganglia, cerebellum, and cerebral cortex: their role in planning and controlling action Cereb Cortex 1995 5 02 95 110 10.1093/cercor/5.2.95 7620294

[106] Iorio-Morin C Fomenko A Kalia S K Deep-Brain Stimulation for Essential Tremor and Other Tremor Syndromes: A Narrative Review of Current Targets and Clinical Outcomes Brain Sci 2020 10 12 925 10.3390/brainsci10120925 33271848 PMC7761254

[107] Dallapiazza R F Lee D J De Vloo P Fomenko A Hamani C Hodaie M Outcomes from stereotactic surgery for essential tremor J Neurol Neurosurg Psychiatry 2019 90 04 474 482 10.1136/jnnp-2018-318240 30337440 PMC6581115

